# Maintaining essential tuberculosis services during the COVID-19 pandemic, Philippines

**DOI:** 10.2471/BLT.21.286807

**Published:** 2021-11-25

**Authors:** Marianne Calnan, Alexander Moran, Hala Jassim AlMossawi

**Affiliations:** a University Research Co., USAID TB Platforms for Sustainable Detection, Care and Treatment Project, Manila, Philippines.; b University Research Co., 5404 Wisconsin Ave, Suite 800, Chevy Chase, MD 20815, United States of America.

## Abstract

**Objective:**

To describe the implementation, use and cost of a phone-based tuberculosis case finding and case management intervention during the coronavirus disease 2019 (COVID-19) pandemic in two regions in the Philippines.

**Methods:**

We implemented this phone-based intervention to maintain tuberculosis treatment support, active case finding and contact investigation efforts in 42 facilities, starting in June 2020. We established a dedicated mobile phone number for each centre and promoted the intervention on different media platforms. We recruited and trained staff members and provided them with tools for screening and patient follow-up. We collected data on tuberculosis screening, diagnosis and treatment initiation for this intervention and three comparator interventions over the same period. We collected data on number and type of calls placed and received. We estimated the additional cost of this intervention compared to the standard of care.

**Findings:**

From October 2020 to September 2021, 14 tuberculosis contact centres, for which complete data were available, identified 43.5% (827/1901) of patients with bacteriologically confirmed tuberculosis enrolled in treatment among all comparator interventions. These centres managed 6187 calls over the same period. The additional cost of implementing and running the centre for 12 months was 398 United States dollars per facility.

**Conclusion:**

The tuberculosis contact centre is a low-technology telehealth intervention which contributed to overall treatment initiation during the COVID-19 pandemic. Additional work should assess the extent to which the contact centre identifies tuberculosis patients previously missed by the health system, regardless of the pandemic.

## Résumé

**Objectif:**

Décrire la mise en œuvre, l'utilisation et le coût de la campagne d'identification et de suivi des cas de tuberculose par téléphone durant la pandémie de maladie à coronavirus 2019 (COVID-19) dans deux régions des Philippines.

**Méthodes:**

Nous avons organisé cette campagne téléphonique afin de poursuivre les efforts d'aide au traitement contre la tuberculose, de détection active des cas et de recherche des contacts dans 42 établissements, en commençant en juin 2020. Nous avons créé un numéro de téléphone mobile propre à chaque centre et fait la promotion de la campagne sur différentes plateformes médiatiques. Nous avons recruté et formé des membres du personnel, puis leur avons fourni des outils de repérage et de suivi des patients. Nous avons récolté des données sur le dépistage de la tuberculose, le diagnostic et le début du traitement pour cette campagne ainsi que pour trois autres initiatives comparables se déroulant sur la même période. Nous avons également rassemblé des informations sur le nombre et le type d'appels passés et reçus. Enfin, nous avons estimé le coût supplémentaire de cette campagne par rapport aux soins habituels.

**Résultats:**

Entre octobre 2020 et septembre 2021, 14 centres de contact pour la tuberculose disposant de données complètes ont identifié, parmi l'ensemble des initiatives comparables, 43.5% (827/1901) de patients qui présentaient une tuberculose avec confirmation bactériologique et avaient entamé un traitement. Ces centres ont géré 6187 appels sur la même période. La mise en œuvre et l'exploitation du centre pendant 12 mois ont engendré un coût supplémentaire de 398 dollars américains par établissement.

**Conclusion:**

Le centre de contact pour la tuberculose est un outil de télémédecine rudimentaire qui a contribué à instaurer un traitement global durant la pandémie de COVID-19. D'autres travaux devraient être menés pour évaluer dans quelle mesure le centre de contact détecte les patients atteints de tuberculose et passés jusque-là à travers les mailles du système de santé, indépendamment de la pandémie.

## Resumen

**Objetivo:**

Describir la aplicación, el uso y el coste de una intervención de búsqueda y gestión de casos de tuberculosis por teléfono durante la pandemia de la coronavirosis de 2019 (COVID-19) en dos regiones de Filipinas.

**Métodos:**

Se aplicó esta intervención por teléfono para mantener los esfuerzos de apoyo al tratamiento de la tuberculosis, la búsqueda activa de casos y la investigación de contactos en 42 centros, a partir de junio de 2020. Se estableció un número de teléfono móvil exclusivo para cada centro y se promovió la intervención en diferentes plataformas de medios de comunicación. Se reclutó y formó al personal y se le proporcionaron herramientas para el cribado y el seguimiento de los pacientes. Se recopilaron datos sobre el cribado, el diagnóstico y el inicio del tratamiento de la tuberculosis para esta intervención y tres intervenciones de comparación durante el mismo periodo. Asimismo, se recopilaron datos sobre el número y el tipo de llamadas realizadas y recibidas. Por último, se calculó el coste adicional de esta intervención en comparación con la atención estándar.

**Resultados:**

De octubre de 2020 a septiembre de 2021, 14 centros de contacto para la tuberculosis, de los que se disponía de datos completos, identificaron al 43.5 % (827/1901) de los pacientes con tuberculosis bacteriológicamente confirmada inscritos en el tratamiento entre todas las intervenciones comparativas. Estos centros gestionaron 6187 llamadas durante el mismo periodo. El coste adicional de la aplicación y el funcionamiento del centro durante 12 meses fue de 398 dólares estadounidenses por centro.

**Conclusión:**

El centro de contacto para la tuberculosis es una intervención de telemedicina de baja tecnología que contribuyó al inicio general del tratamiento durante la pandemia de la COVID-19. Otros estudios deberían evaluar en qué medida el centro de contacto identifica a los pacientes con tuberculosis que el sistema sanitario no había detectado antes, con independencia de la pandemia.

## ملخص

**الغرض:**

وصف كيفية تنفيذ اكتشاف حالة سل عبر الهاتف واستخدام هذا الاكتشاف وتكلفته، وكذلك تدخل إدارة الحالة أثناء جائحة مرض فيروس كورونا 2019 (كوفيد 19) في منطقتين في الفلبين.

**الطريقة:**

قمنا بتنفيذ هذا التدخل عبر الهاتف للحفاظ على دعم علاج مرض السل، واكتشاف الحالات النشطة، وجهود التحقيق مع جهات الاتصال في 42 مرفقًا، بدءًا من يونيو (حزيران) 2020. قمنا بتخصيص رقم هاتف محمول لكل مركز، وعززنا التدخل على منصات مختلفة للوسائط. قمنا بتوظيف وتدريب أفراد فريق العمل، وزودناهم بأدوات للفحص ومتابعة المرضى. قمنا بجمع بيانات حول فحص مرض السل وتشخيصه، وبدء العلاج لهذا التدخل وثلاث تدخلات مقارنة خلال نفس الفترة. كما قمنا بجمع بيانات عن عدد ونوع المكالمات التي تم إجراؤها وتلقيها. قدرنا التكلفة الإضافية لهذا التدخل مقارنة بمستوى الرعاية.

**النتائج:**

من أكتوبر (تشرين الأول) 2020 إلى سبتمبر (أيلول) 2021، قام 14 مركزاً للاتصال خاص بمرض السل، وتوفرت عنها بيانات كاملة، بتحديد 43.5% (827/1901) من المرضى المصابين بالسل والمؤكدة حالاتهم عن طريق الفحص البكتريولوجي، والمسجلين في العلاج من بين جميع التدخلات المقارنة. واستطاعت هذه المراكز إدارة 6187 مكالمة خلال نفس الفترة. وبلغت التكلفة الإضافية للتنفيذ وتشغيل المركز لمدة 12 شهرًا، 398 دولارًا أمريكيًا لكل مرفق.

**الاستنتاج:**

مركز الاتصال الخاص بمرض السل هو تدخل صحي عن بعد منخفض التكنولوجيا، ساهم في بدء العلاج بشكل عام خلال جائحة كوفيد 19. يجب أن يقوم العمل الإضافي بتقييم مدى تحديد مركز الاتصال لمرضى السل الذين تخلفوا عن النظام الصحي سابقًا، بغض النظر عن الجائحة.

## 摘要

**目的:**

旨在描述菲律宾两个地区新型冠状病毒肺炎 (COVID-19) 疫情期间基于电话的结核病病例筛检和病例管理干预措施的实施、使用和成本。

**方法:**

我们自 2020 年 6 月在 42 个机构中实施了这项基于电话的干预措施以便维持结核病治疗支持、积极的病例筛检和联络调查工作。我们为每个中心建立了专用的手机号码，并在不同的社交媒体上宣传推广这项干预措施。我们招募并培训了工作人员并为他们提供工具以开展筛检和患者追踪。我们针对这项干预措施和同一时期的三项比较措施收集了结核病筛检、诊断和治疗起始数据。我们收集了关于拨打和接听的电话数量和类型数据。我们评估了这项干预措施较之标准护理的额外费用。

**结果:**

自 2020 年 10 月到 2021 年 9 月，在所有比较干预措施中，14 家拥有完整数据的结核病联络中心识别了 43.5% (827/1901) 经细菌学检验确诊患有结核病的患者登记接受治疗。这些中心在上述同一时间段内记录了 6187 次通话。持续 12 个月实施和运行中心所产生的额外成本是每个中心 398 美元。

**结论:**

结核病联络中心是一种技术含量低的远程医疗干预措施，有助于在新型冠状病毒肺炎疫情期间实现全面的起始治疗。还应该进一步评估联络中心在多大程度上识别了之前被卫生系统遗漏的患者，无论是否处于疫情期间。

## Резюме

**Цель:**

Описать внедрение, использование и затраты на выявление случаев туберкулеза путем телефонных опросов, а также меры по ведению больных во время пандемии коронавирусной инфекции 2019 года (COVID-19) в двух регионах Филиппин.

**Методы:**

Авторы внедрили эту дистанционную меру для оказания поддержки при лечении туберкулеза, раннего выявления заболевания и отслеживания контактов с заразными больными в 42 учреждениях начиная с июня 2020 года. Была открыта линия мобильной связи для каждого центра, и соответствующие телефонные опросы были внедрены на различных медийных платформах. Были наняты и обучены сотрудники, которым предоставили инструменты для скрининга и последующего наблюдения за больными. Авторы собрали данные о скрининге, диагностике и начале лечения туберкулеза для этой меры и трех мер сравнения за тот же период. Авторы собрали данные о количестве и видах совершенных и полученных телефонных звонков. Они оценили дополнительные расходы этой меры по сравнению со стандартами медицинской помощи.

**Результаты:**

С октября 2020 года по сентябрь 2021 года в 14 контактных центрах борьбы с туберкулезом, по которым были доступны полные данные, было выявлено 43.5% пациентов (827/1901) с бактериологически подтвержденным туберкулезом, включенных в лечение на основании всех мер сравнения. Эти центры обработали 6187 звонков за тот же период. Дополнительные расходы на внедрение и эксплуатацию центра в течение 12 месяцев составили 398 долларов США на одно учреждение.

**Вывод:**

Контактный центр борьбы с туберкулезом — это достаточно простая мера телемедицины, которая способствовала общему началу лечения во время пандемии COVID-19. Независимо от пандемии, следует провести дополнительные работы и оценить степень выявления контактным центром больных туберкулезом, которых ранее система здравоохранения не учитывала.

## Introduction

The coronavirus disease 2019 (COVID-19) pandemic has challenged modern public health practices because the response to the pandemic has introduced several health systems pressures related to economic resources, human resources and essential service delivery.^1^^,^^2^ For example, tuberculosis case finding, treatment initiation and treatment adherence have been greatly affected during the pandemic.^3^^,^^4^ The *Global tuberculosis report 2021* reported an 18% reduction in new tuberculosis diagnoses, from 7.1 million in 2019 to 5.8 million in 2020, and an increase in tuberculosis deaths among human immunodeficiency virus negative people, from an estimated 1.2 million deaths in 2019 to 1.3 million in 2020.^4^ While a decrease in tuberculosis case finding has been observed globally, the impact of the COVID-19 pandemic on tuberculosis services has been particularly acute in the Western Pacific Region.^4^^,^^5^ In the Philippines, estimates suggest that aggregate weekly tuberculosis notification decreased by 79% (from 15 to 22 March 2020) after the imposition of travel restrictions, community quarantine, shifting diagnostic and clinical resources from tuberculosis to COVID-19 and limited contact tracing of tuberculosis patients.^5^^,^^6^ These mitigation strategies also led to discontinuation of active tuberculosis case finding and reduction in demand for private providers’ tuberculosis services.^5^^,^^7^

In June 2020, the World Health Organization (WHO) Regional Office for the Western Pacific organized a meeting on tuberculosis services for representatives from China, Malaysia and the Philippines. The participants identified priority actions for restoration of tuberculosis services, including online consultations for individuals with presumptive tuberculosis, digital platforms and laboratory connectivity solutions for rapid results delivery, digital adherence interventions, digital mechanisms for managing adverse drug reactions and innovative digital health education approaches.^5^

Interventions to increase the reach of tuberculosis services in the Philippines have been in place before the pandemic. For example, in October 2019, the United States Agency for International Development (USAID) TB Platforms for Sustainable Detection, Care and Treatment Project implemented a phone-based tuberculosis contact centre in a single facility in Marawi City, the capital city of Lanao del Sur province in Bangsamoro Autonomous Region. Implementation in this facility was paused following a polio outbreak in the area that first began in September 2019 and eventually required the redistribution of health-care workers to polio outbreak management efforts.^8^ To mitigate the impact of shifting priorities, constrained resources and limited mobility on tuberculosis services during the COVID-19 pandemic, we implemented this contact centre intervention across two regions in the Philipines.^5^^,^^6^^,^^9^ Here, we describe the implementation of the centres in health-care facilities and the contribution of these centres to tuberculosis case-finding efforts during the COVID-19 pandemic. We assess indicators throughout the tuberculosis care cascade, including screening, identification of people with presumptive tuberculosis, diagnosis and treatment enrolment. In addition, we present data on use of the centre for services like adherence support, which includes reporting of adverse drug reactions. 

## Methods

### The intervention

The centre is a low-technology phone-based telehealth intervention which is based at individual facilities with a tuberculosis basic management unit. The intervention aims to maintain essential tuberculosis services by leveraging existing mobile network infrastructure. The freely accessible phone-based services focus on contact screening services, diagnostic results delivery, adherence and treatment management and adverse drug reactions, and align to WHO priority actions for restoring tuberculosis services and serve as additional points of contact to the health system.^5^

Anyone in the facility catchment area can call the centre during the opening hours (that is, 5 days a week from 07:00 to 17:00) for information on tuberculosis services. Health-care providers at the centre call tuberculosis patients receiving services at a centre facility, close household contacts, people presumed to have tuberculosis after screening at the facility, and make subsequent referral calls. For patients younger than 18 years, parents or legal guardians are eligible to use the centre on behalf of the patient. Awareness building activities in the communities surrounding centre facilities – including radio advertisement, print media, billboards and social media – encourage community members to call the centre.

For individual calls, the provider records all data on a patient contact form, which includes demographic information, reason for the call, notes, actions taken because of the call, tuberculosis symptom screening results, risk factors for special risk groups and follow-up steps, including facility referrals. Referrals are also documented on a call summary log sheet so health-care workers can track referral completion. Health workers at the centres call referral facilities after 5 days to cross-check whether a patient has arrived and received services. 

The providers call all contacts listed on the tuberculosis patient card for symptom screening. For each call, the provider notes demographic details about the contact on a patient contact form and documents that the call is for tuberculosis screening. The symptom screen is conducted during the phone call. For people who are identified as having presumptive tuberculosis, the provider refers patients to nearby facilities and follows up with the patient and the facility to ensure the referral and subsequent tuberculosis testing have been completed. After completion, the patient contact form is annotated to document the completed referral.

Patient contact forms and call summary log sheets are paper based, but the nurses at the centre digitally enter data from the call summary log sheet for submission to the project in deidentified, aggregated form. Paper forms are stored in folders in locked cabinets in the tuberculosis unit with tuberculosis registers and are accessible only to the staff members of the clinic. Summary data are transferred to and stored in the project’s secure cloud server and do not contain any personally identifying information. 

**Fig. 1:**
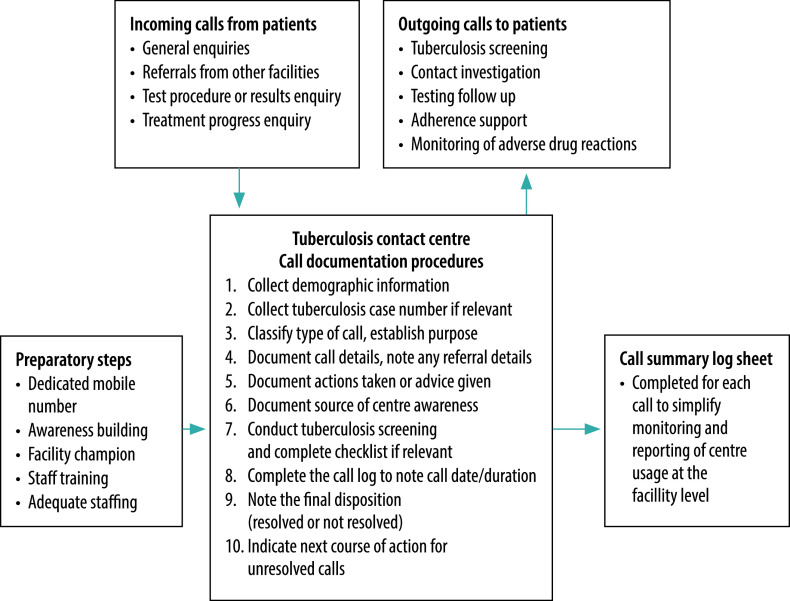
Implementation framework of facility-level tuberculosis contact centre, Philippines, 2020

Fig. 1 shows how the centres are operating and also includes the implementation steps. 

### Implementation

In response to COVID-19 community quarantine enforced on 15 March 2020, the implementation expanded to 14 facilities in Region IV-A starting in June 2020 and 28 facilities in Region III starting in August 2020. We selected these sites purposively, including both rural health units and city health offices, prioritizing sites with high patient volume, staff willingness to operate the contact centre and leadership commitment to sustain the initiative. 

We conducted preparatory steps at each contact centre before implementation began. These steps included establishing a dedicated mobile phone number for the contact centre at the facility, promoting the centre using radio advertisements, print media, billboards and social media, designating staff members for operation, training of staff members and recruitment of sufficient staff members in the facility for implementation and providing tools for documenting calls, screening and patient follow-up. Training included a 3- to 4-hour online orientation on initiating and operating the contact centre. We prioritized staff members from the tuberculosis clinic for training, but other cadres including nurses in the outpatient department could also attend. To improve the process, the project staff members regularly observed how the patients were identified from the tuberculosis register, how the calls were made and how the documentation was done. Based on this information, post-training feedback was given a week after the training session and was subsequently provided monthly or bimonthly.

During the training and start-up activities, we identified an implementation champion for each centre. This champion is a health-care worker who is involved with the centre’s implementation and who can encourage patients to use the centre. The champion also serves as a point of contact for the project to monitor implementation.

### Data and analysis

We used secondary cross-sectional programmatic data collected through the centres to analyse the contribution of the centres to tuberculosis care. The primary outcomes for this descriptive analysis include tuberculosis cascade of care outcomes (screening, presumptive identification, diagnosis and treatment initiation) to measure contribution of the centres to tuberculosis services in comparison to other comparator interventions.

To describe the uptake and interest in the intervention, we report on centre service use, including incoming and outgoing calls by category. Screening data were available for all 42 centres from October 2020 to September 2021. Over the same period, full care-cascade data were only available in 14 facilities in Region IV-A, which we assessed in a subgroup analysis. We used the results from an interim internal assessment of programmatic data to report on contact centre usage from October 2020 to September 2021 for the 14 centres in Region IV-A.

To compare with other tuberculosis case-finding interventions, including active, enhanced and intensified case finding, we obtained data from the integrated tuberculosis information system in summary form for the period October 2020 to September 2021. Active and enhanced case finding are two community-based case-finding approaches in which health-care workers provide tuberculosis services in the community. Active case finding focuses on symptom screening and chest X-ray services provided on-site, with subsequent sputum sample provided or collected on-site and referred for processing. Enhanced case finding focuses on symptom and risk factor screening in the community and subsequent referral to health facilities for additional screening, sputum collection and other services as indicated. Intensified case finding (that is, FAST, finding cases actively, separating safely and treating effectively) is a hospital-based universal case-finding strategy which seeks to identify unsuspected tuberculosis in hospital settings through universal screening of people entering hospitals, separation of people with presumptive tuberculosis, prompt testing with a molecular diagnostic test and rapid initiation of effective anti-tuberculosis therapy. This intervention has been previously described in detail in a similar setting.^10^ Since we implemented the contact centres in intensified case finding implementing sites, we distinguished between patients who presented at the facilities with a referral from the contact centres and patients presenting with referrals from other case-finding activities or without a referral. 

To estimate the additional cost of the centres, we used data on the cost of planning meetings, conducting training, buying mobile phones, buying initial and recurring radio airtime and conducting awareness activities. We calculated the implementation cost for 12 months for a single facility and present results for the overall cost among all 42 facilities. We did not provide any reimbursement, either for the use of the contact centre or the training. 

We did not seek ethical approval for this analysis as all data were collected for programmatic, quality improvement purposes in deidentified and summary form. Additionally, because this intervention was designed as a public health service implemented in collaboration with the Philippine government and not a research study, we did not collect consent from participants for using the centre, nor was there any official recruitment into the contact centre.

## Results

As of October 2021, all 42 facilities had an active contact centre. The centres screened 10 976 individuals from October 2020 to September 2021 and accounted for 1.2% (10 976/887 495) of all screening activities among comparators.

**Fig. 2:**
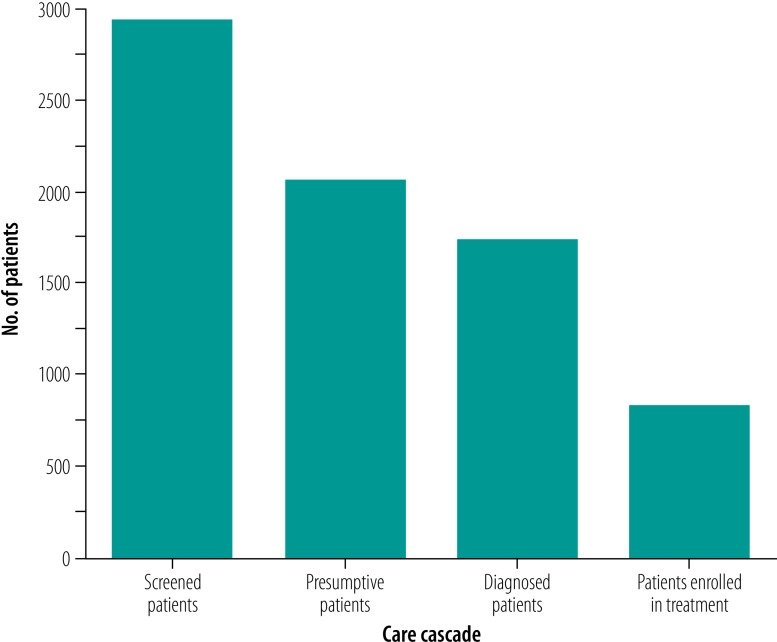
Tuberculosis care cascade at tuberculosis contact centre, Philippines, October 2020–September 2021

Note: Data from 14 facilities in administrative Region IV-A.

**Fig. 3:**
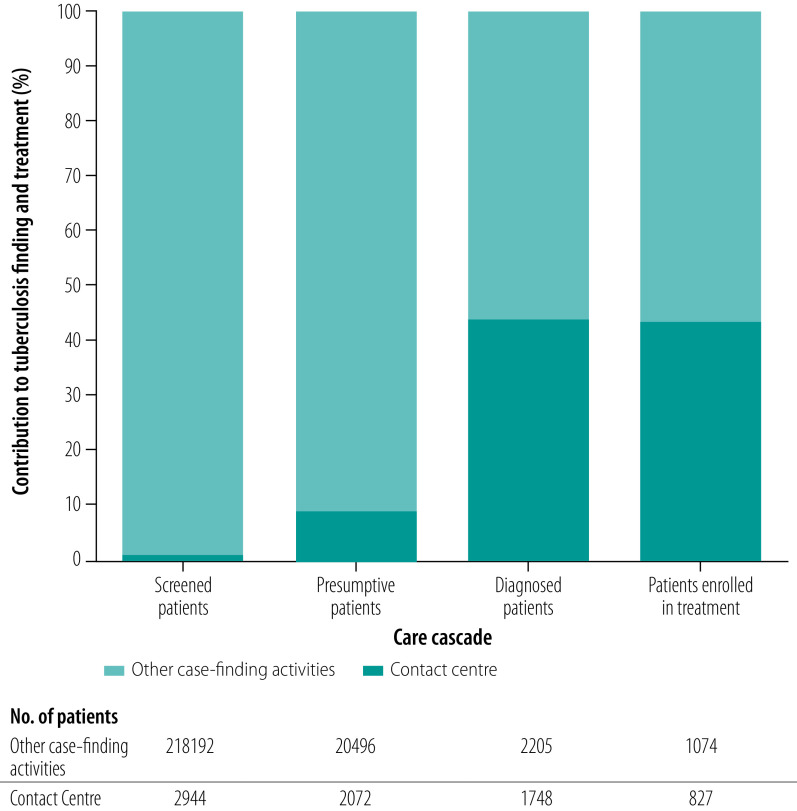
Tuberculosis contact centre contribution to tuberculosis cascade of care indicators, Philippines, October 2020–September 2021

Note: Data from administrative Region IV-A. Contact centre data cover 14 facilities. Other case-finding activities are active, enhanced and intensified case finding.

For 14 facilities with available data on the care cascade, 70.4% (2072/2944) of the people screened were presumed to have tuberculosis (Fig. 2). These centres accounted for identifying 9.2% (2072/22 568) of all people presumed to have tuberculosis among all interventions in Region IV-A. The centres identified 44.2% (1748/3953) of all confirmed tuberculosis patients among all interventions and 43.5% (827/1901) of patients with bacteriologically confirmed tuberculosis enrolled in treatment were identified through the centres (Fig. 3).

We assessed all available use data in Region IV-A. A total of 6187 calls were recorded from October 2020 to September 2021 (342 incoming and 5845 outgoing calls) among the 14 facilities. Outgoing calls often covered multiple issues (Box 1). Incoming calls were not available in disaggregated form for this analysis.

Box 1 **Use of selected services at the tuberculosis contact centre, Philippines, October 2020 to September 2021**

**Incoming calls: 342**

**Outgoing calls**

- Treatment monitoring: 1504
- Schedule for testing or provider-initiated counselling and testing: 1256
- Treatment enrolment: 1112
- Screening, counselling and consultation: 750
- Test results update: 668
- Sputum follow-up: 572
- Referral coordination: 512

The total cost of implementing the contact centre in 42 facilities for 12 months was 25 074 United States dollars (US$). Costs during start-up activities were the main additional costs outside of normal programmatic costs and staff time. The start-up costs were limited to procurement of equipment including mobile phones, radio airtime, training activities and awareness costs. Recurring costs included ongoing radio airtime. Table 1 shows start-up and recurring costs for the first year of implementation. Using the 14 facilities in Region IV-A with complete cascade data as an illustration, the total 1-year cost for implementing the centres in these facilities is about US$ 5572. Given that there were 827 tuberculosis treatment initiations from these 14 centres, the crude cost per centre-identified patient started on treatment is about US$ 7 additional to the cost of standard of care. 

**Table 1:** Start-up and recurring tuberculosis contact centre implementation costs, Philippines, 2020–2021

Item	Single facility, US$	All facilities, US$ (*n* = 42)
**Startup^a^**	326	20 538
Planning meetings	10	630
Mobile phone	160	10 080
Initial radio airtime allowance	10	630
Training	76	4 788
Awareness: leaflets	70	4 410
**Recurring^b^**	72	4 536
Radio airtime	72	4 536
**Total implementation cost^c^**	**398**	**25 074**

US$: United States dollars.

^a^ One-time cost.

^b^ Monthly recurrence, presented for 12 months of implementation.

^c^ For 12 months.

## Discussion

The contact centre intervention is a targeted, low-cost patient support and case-finding strategy which offers a low-technology telehealth solution for maintaining engagement with essential tuberculosis services. The contribution of the centres to the efforts of screening and identifying people with tuberculosis increased throughout the care cascade, while the contribution of patients enrolled in treatment were similar to the share of identifying people with confirmed tuberculosis. The majority of calls were outgoing calls from health-care workers to current patients or close household contacts, while a small share of calls were people who called the centres about treatment or had general enquiries. Finally, the intervention cost was about US$ 7 per person started on treatment through the centres. Similar work suggests that active case-finding strategies compared to standard passive case-finding interventions have an estimated cost–effectiveness of US$ 330 per disability-adjusted life year averted.^11^^–^^14^

The initial contact centre implementation in Marawi City which began in 2019 was designed for a post-conflict setting with a specific need for low-cost, low-technology telehealth solutions which do not rely on internet for utilization. Upon the expansion and adoption of the intervention to respond to COVID-19 community quarantine, the centre stands as a unique intervention in the Philippines for maintaining essential tuberculosis services. While the Philippine government originally used a similar cell phone-based system for COVID-19 contact tracing, the StaySafe app was later adopted to manage contact tracing activities in preference to the lower-technology alternative.^15^^–^^17^

United Nations Habitat estimates that in the Philippines, nearly 43% of the urban population live in slums.^18^ The urban poor often have limited access to necessities and limited disposable income, likely reducing access to mobile internet or to smartphones in general. Because the urban poor are considered to be at highest risk for poor outcomes from tuberculosis, a system which does not require internet or smartphone access was an essential consideration to ensure wide access.^19^ Reports show that the community quarantine and travel restrictions were more strictly enforced in administrative regions III, IV-A and National Capital, which have a large population of the urban poor. These restrictions may have reduced access to essential tuberculosis services and access to high-technology telehealth services among the urban poor population. These issues are exposing a gap between urban populations in access to services, maintenance on tuberculosis treatment and contact tracing.^20^^,^^21^ As a selective screening intervention, the centres aim to fill this access gap, exacerbated by the COVID-19 pandemic, by identifying people at high risk for tuberculosis who would otherwise be missed by the health system rather than acting as a population-level screening intervention.

Despite the success of the centres in maintaining essential tuberculosis services, we encountered certain implementation challenges. First, in many cases there were insufficient human resources to staff the centres, especially during high call traffic times or outside regular office hours. Second, patients in rural areas had occasional difficulties interacting with the centres due to poor cellular signals or insufficient funds to make phone calls. Finally, an inherent limitation to the phone-based centres is that patient status cannot be assessed visually by the health-care worker. To mitigate the impact of these challenges, we ensured that multiple facility staff were trained on centre implementation so a larger pool of available health-care workers could participate. Unfortunately, we could not assess the extent to which community members who wanted to call the centres were unable to do so.

The cross-sectional, aggregate and deidentified data used to analyse the contribution of the centres have limitations. Data collection challenges in certain facilities limited our ability to analyse the entire cascade of care outside of Region IV-A, and these challenges highlight the need to improve data collection and reporting processes as implementation continues. Additionally, the underlying risk distribution for tuberculosis is probably different among the target population of the comparator interventions, which could explain differences presented in our results.

The contact centre intervention provides a strategy to reach the urban poor and rural populations, who have less access to health-care services and to technical solutions, such as internet and mobile applications. With the centres’ specific scope, ease of use for patients and clear reporting mechanisms, the centres benefit patients, providers and the national tuberculosis programme in the Philippines. The system rationally uses human and capital resources, ensuring that many people benefit from a relatively small investment. Additionally, the system performs well as a targeted screening approach, identifying a high proportion of people presumed to have tuberculosis and exhibiting high tuberculosis case yield. Ongoing work should consider whether the selective approach and high yield is entirely attributable to community quarantine during the COVID-19 pandemic, or whether a proportion of centre users are people who would have otherwise been missed by the health system regardless of the pandemic.

After the implementation of the contact centres, the Philippine health department released the *National tuberculosis program adaptive plan* on 9 November 2020, which guided continuity in delivering essential tuberculosis services during the COVID-19 pandemic.^22^ The plan recommends the infection control adaptations of tuberculosis screening, incorporation of tuberculosis testing with COVID-19 testing, integration of tuberculosis screening and testing with COVID-19 vaccination activities and multiweek prescriptions of anti-tuberculosis drugs.

The contact centre intervention is a sustainable case-finding solution. Participating facilities contribute resources to support centre start-up, including staffing and space for the centre. After initial start-up, sites cover the costs for printing patient forms and – in certain cases – for ongoing radio airtime costs. We expect that facilities will support all operational costs of the centres by the planned closeout of the USAID TB Platforms for Sustainable Detection, Care and Treatment Project in 2023. Finally, the project encourages the application of this phone-based model to other programmes including sexual and reproductive health, other infectious diseases and noncommunicable diseases (especially diabetes and hypertension). The intervention has the possibility of reducing barriers to care among those experiencing stigma, people who may have insufficient time or funds to reach a health facility in person or people who prefer to communicate with health providers via phone instead of text messaging or a mobile app. As facilities reopen and in-person care resumes, the tuberculosis contact centres will remain an essential intervention for tuberculosis screening, contact investigation and treatment adherence.
